# The era of histoplasmosis in Brazilian endemic mycoses

**DOI:** 10.1016/j.lana.2021.100037

**Published:** 2021-08-12

**Authors:** Diego R. Falci, Daiane F. Dalla Lana, Alessandro C. Pasqualotto

**Affiliations:** 1 Pontificia Universidade Catolica do Rio Grande do Sul, Porto Alegre, Brazil; 2 Hospital de Clinicas de Porto Alegre, Porto Alegre, Brazil; 3 Universidade Federal de Ciencias da Saude de Porto Alegre, Porto Alegre, Brazil; 4 Santa Casa de Misericordia de Porto Alegre, Porto Alegre, Brazil

Not long ago, most of the literature on endemic mycoses in Brazil focused on paracoccidioidomycosis. With changes in agricultural practices this infection is now reduced in frequency [1] and another fungal disease has received increased attention: histoplasmosis. Not only because *Histoplasma capsulatum* is hyper endemic in the Brazilian territory but mostly because substantial gaps exist in the Brazilian HIV program. Despite having a successful policy of free antiretroviral treatment to people living with HIV (PLHIV), opportunistic infections (OIs) remain frequent in Brazil due to retention in care issues, which are associated to a vicious circle that includes social inequities, mental diseases, drug abuse, and poor education. Also, a significant proportion of patients are ‘late AIDS presenters’, being diagnosed with HIV with an OI already in place. Individuals with advanced AIDS are prone to severe disease caused by *H. capsulatum* and have high case-fatality rates. Barriers to the diagnosis of histoplasmosis and access to optimal antifungal treatment still exist in a country in which prognosis of histoplasmosis in PLHIV is poor. Although being the most prevalent endemic mycosis in the Americas [2], histoplasmosis remains a neglected disease in Brazil.

Diagnosis of histoplasmosis can be tricky, with microscopy requiring considerable expertise. Histopathological findings are also subject to errors, since *H. capsulatum* can be mistaken for many other pathogens*.* A proven diagnosis can be achieved by culture, but the fungus can take up to 6 weeks to grow; both histopathology and culture invariably need a biopsy to get access to tissues (e.g. skin, bone marrow), which is frequently difficult to obtain. Blood cultures are not reliable (aside from the poorly available lysis-centrifugation method), and serology has unacceptable low sensitivity in immunocompromised patients – apart from the promising Western Blot methodology, but experience is still limited. At the moment, diagnosis is best performed using one of the urinary *Histoplasma* antigen detection assays. These are rapid, non-invasive, and non-culture based diagnostic tools, with excellent sensitivity and specificity, and elevated negative predictive value. Two of these assays were recently validated: ‘Clarus Histoplasma GM Enzyme Immunoassay’ (Immy Diagnostics, Norman, OK, USA) [3], and the ‘MiraVista *Histoplasma* Urine Antigen LFA’ (Miravista Diagnostics, Indianapolis, IN, USA). [4] Cost per assay of these tests may be as low as $10. Still in Brazil only 16% of reference centres have access to *Histoplasma* antigen detection, demonstrating how the country is regarding optimal care of histoplasmosis in PLHIV. PCR is an encouraging diagnosis tool, but its use and availability are limited to research centres.

It seems clear that proper and timely diagnosis can revert the rampant killing of AIDS patients by *H. capsulatum*. In a modelling study using prevalence of histoplasmin positive reactions and the incidence of HIV, authors estimated that 1.1-6.7K annual deaths would occur due to histoplasmosis, considering different scenarios of 10-60% case-fatality rates. [5] The lethality rate varied largely in the Americas: in North America, where there are sufficient resources and the diagnosis of histoplasmosis can be made as early as 2-5 days, case-fatality was ∼10%. Conversely, in the absence of antigen detection, diagnosis of histoplasmosis in Latin America typically takes 14-21 days (time for cultures to turn positive) and case-fatality is above 40% [6]. Brazil is positioned in the upper range of mortality rates in the continent. From an estimate of 13,000 annual AIDS deaths, histoplasmosis may have a significant proportion of deaths, with numbers similar to those of tuberculosis. [5] In a study of 208 patients with disseminated histoplasmosis, at a reference in the Northeast region of Brazil from 2006-2007, case fatality rate was 42.3%. [7] An even higher mortality rate (53.0%) was observed in a study from Goias, in the Central-West Region of Brazil. [8]

Considering that diagnostic tests are commercially available to facilitate management of histoplasmosis, to reduce mortality and to potentially save thousands of lives per year, what seems to be the point? Lack of disease awareness is certainly an issue, so scientists should keep saying it loud: histoplasmosis is a rampant killer of PLHIV in Brazil. Second, barriers to systematic antigen detection implementation in the Brazilian HIV National Program must be surpassed, in agreement with the recent Manaus Declaration, about histoplasmosis in the Americas and the Caribbean. [9] The World Health Organization (WHO) has issued guidelines for managing advanced AIDS, recommending a package of interventions for these patients targeting rapid diagnosis of OIs and initiation of antiretroviral therapy. Even though histoplasmosis diagnosis is not yet included in this package, WHO has recently included *H. capsulatum* antigen detection in the Second WHO Model List of Essential In Vitro Diagnostics. [10] Therefore, there is an urgent need to incorporate *Histoplasma* antigen detection in Brazilian HIV Program, and this must include private medicine as well. Even though <30% of Brazilians are attended in the private sector, incorporation of *Histoplasma* diagnostics by the National Agency for Supplementary Health list of exams and procedures would expand access to diagnostic tests to PLHIV. Both tests from Miravista and Immy Diagnostics are already approved by the Brazilian Health Regulatory Agency and can be marketed in Brazil.

Regarding antifungal therapy, amphotericin B has been the antifungal agent of choice for induction therapy of moderate to severe disseminated histoplasmosis, with the sequential use of itraconazole as maintenance therapy. Considering the different amphotericin B formulations, liposomal amphotericin B (L-AmB) is known to achieve higher drug concentrations in the reticuloendothelial system and to be considerably less nephrotoxic, although being more expensive than the parental drug amphotericin B deoxycholate (d-AmB). [11] In Brazil, most AIDS patients with disseminated histoplasmosis are treated with the most toxic of the amphotericin B formulations, d-AmB, despite the publication of a clinical trial 19 years ago demonstrating the superiority of L-AmB over d-AmB in disseminated histoplasmosis in AIDS. [11] Even though Brazil has a large national program of mycoses control which provides amphotericin B lipid complex to patients with confirmed fungal infections, AIDS patients with histoplasmosis are excluded from this program, since these are (in theory) under the radar of Brazilian HIV Program – that in reality has no budget to L-AmB or any lipid amphotericin B formulation. Assuming 2,340 annual cases as in Adenis et al. [5], and the differences in survival demonstrated in the clinical trial by Johnson and cols. [11], 164 lives could be saved every year in Brazil if all patients diagnosed were treated with L-AmB instead of d-AmB. And these numbers are probably underestimated. To validate alternative uses of L-AmB in disseminated histoplasmosis in AIDS, we are currently evaluating the clinical use of single high doses of L-AmB (Clinical Trials number NCT04059770). If that proves to be safe and efficient, improved access to L-AmB may occur, by reducing the overall costs associated with antifungal treatment.

In such a diverse and continental country, it is not a surprise that histoplasmosis is not equally distributed in Brazil. For instance, histoplasmosis was diagnosed in >40% of febrile hospitalized AIDS patients in Central-Northeast Brazil, whilst prevalence rates were below 10% in South and Southeast Brazil [12] (Figure 1). Other studies have come with similar figures: 41% prevalence in Ceara State (Northeast Brazil) [13], and 7.5% in AIDS patients from Sao Paulo (the largest Brazilian city, located in Southeast Brazil) [14]. Therefore, most of the burden of histoplasmosis occur in the poorer and underdeveloped regions of the country. It is important to reinforce that the current knowledge on histoplasmosis in Brazil relies on a scenario of an underdiagnosed disease and the real burden of histoplasmosis and its associated mortality remain underestimated.

**Figure 1:**
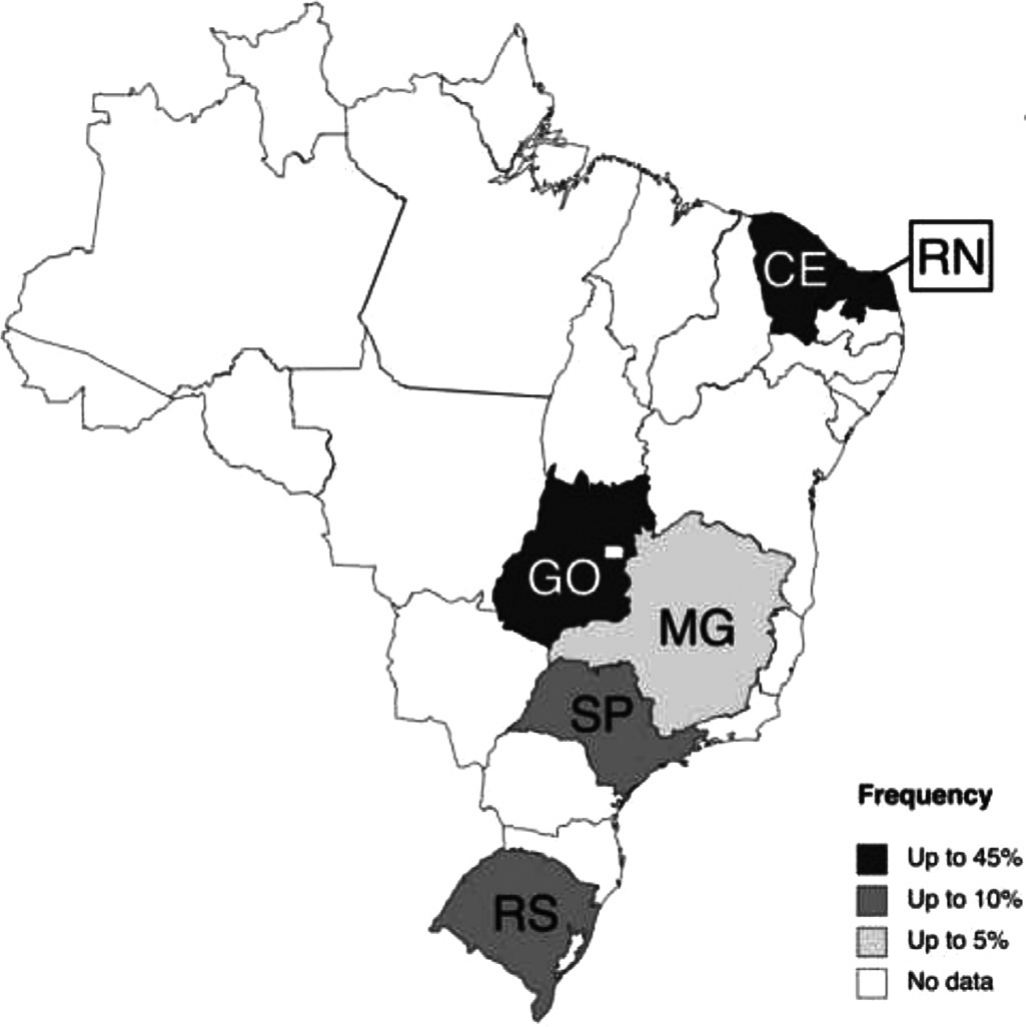
Frequency of probable/proven histoplasmosis in PLHIV in Brazil, according to state. Only studies (n=9) evaluating PLHIV admitted to hospital, reporting frequency of histoplasmosis and in which ≥10 patients were evaluated were included (references 12-20). Case reports, case series and autopsy studies were not included. Legend (counties): CE, Ceara; MG, Minas Gerais; RS, Rio Grande do Sul; RN, Rio Grande do Norte; SP, Sao Paulo; GO, Goias [12], [13], [14], [15], [16], [17], [18], [19], [20].

Despite being a frequent and severe problem to AIDS patients in Brazil, histoplasmosis appears to be an unknown disease to health care policy makers in the largest Latin American country. Governmental data about the condition is not reliable, since it is not a notifiable disease. With very limited access to *Histoplasma* antigen detection, diagnosis of disseminated histoplasmosis still relies on slow-growing cultures, and in methods which are associated with limited sensitivity such as microscopy, histopathology and serology. Patients are still treated with the 60-year-old toxic compound d-AmB, and access to L-AmB remains very limited. It is time to act.

## Contributors

DRF drafted the manuscript; DFDL wrote the part on antifungal therapy; ACP conceived the idea and supervised all work. All authors reviewed and approved the final version of the manuscript.

## Declaration of interests

DRF has received speaker honoraria on behalf of Gilead, United Medical, MSD, and Pfizer. He has participated in the safety advisory board of GSK and received research material from IMMY. DFDL has no conflict of interest to declare. ACP has consulted and/or given paid talks for Gilead, IMMY, United Medical, Pfizer, Teva and MSD, as well as received research grants from Gilead, and research material from IMMY.
